# Impact on Students of the Act–Belong–Commit Mentally Healthy Schools Framework

**DOI:** 10.3390/children10030548

**Published:** 2023-03-14

**Authors:** Robert J. Donovan, Catherine F. Drane, Julia Anwar-McHenry

**Affiliations:** 1 School of Human Sciences, University of Western Australia, Perth, WA 6009, Australia; 2 Future of Work Institute, Curtin University, Perth, WA 6845, Australia; 3 Western Australian Department of Education, Albany, WA 6330, Australia

**Keywords:** mental health promotion, health-promoting schools, Act–Belong–Commit

## Abstract

Schools can have a significant role in affecting the mental health and wellbeing of both students and staff, with considerable implications for society as a whole. Hence, there is a need for school-based interventions to both assist those experiencing mental health problems and to implement activities and policies that facilitate the enhancement and maintenance of good mental health. Unlike most school mental health interventions that are focussed on, and specific to, the school setting, the Act–Belong–Commit Mentally Healthy Schools Framework is based on the principles of the Act–Belong–Commit community-wide general population mental health promotion campaign, which has been adapted to the school setting via the World Health Organisation’s Health Promoting Schools Framework. The Mentally Healthy Schools Framework is a whole-school approach to enhancing both student and staff mental health. This paper reports the findings of a preliminary impact survey administered to students after the adoption of the Framework in a number of primary and secondary schools in Western Australia. Students from two schools that had only recently adopted the Framework completed a “Baseline” questionnaire, and students from three schools that had been implementing the Framework for at least 17 months completed a “Follow-up” questionnaire. The results suggest that the Mentally Healthy Schools Framework, adapted from a community-wide campaign, can have a positive impact on students in terms of increasing openness about mental health, increasing awareness of behaviours conducive to good mental health, and increasing engagement in behaviours to improve their mental health. Such positive impacts have clear implications not only for prevention of mental disorders, but for academic achievement, employment, and overall contribution to society.

## 1. Introduction

Mental health is defined by The World Health Organisation [1] as: “a state of wellbeing in which the individual realises his or her own abilities, can cope with the normal stresses of life, can work productively and fruitfully, and is able to make a contribution to his or her community”. As outlined by Koushede and Donovan [2], an individual’s mental health affects their thinking capacity and hence their ability to learn, which in turn affects educational achievement and opportunities for employment. Mental health issues are also significant risk factors for unintentional and intentional injury and the risk of social isolation [1]. Additionally, as noted by Bourke [3], given the substantial period of their lives spent at school, it is self-evident that their experiences at school would have a considerable influence on their wellbeing, and would impact on both overall behaviour and academic achievement [4]. It is also notable that the second principle of the 2012 Perth Charter for the Promotion of Mental Health and Wellbeing states that, “the foundations of social and emotional wellbeing develop in early childhood and must be sustained throughout the lifespan” [5].

Hence, given that many mental health problems and disorders begin in childhood or adolescence [6,7,8,9,10], and that mental health problems adversely affect behaviour and academic performance, there has been an increasing acknowledgement worldwide that schools are an important setting for the prevention of mental health problems [11,12]. This has resulted in an increasing number of mental health and wellbeing school interventions in Australia and countries around the globe [13,14,15,16,17,18,19]; whilst some interventions are broad-based and implemented internationally [20], most interventions are usually specific to the school setting rather than part of a broader, population-wide mental health initiative, and, as indicated in Liao, et al. [15], are often focussed on mental illness awareness raising and encouraging help seeking rather than emphasising positive mental health and building resilience. In a reflection of an emphasis on mental illness rather than positive mental health, many interventions are led by counsellors rather than the teachers [17]. Further, as noted by Cavioni et al. [21], most focus only on students and are not concerned with teachers’ mental health. Hence, in this paper we report on the impact of a whole-school positive mental health initiative based on a community-wide positive mental health promotion campaign, largely led by the teachers.

### 1.1. The Act–Belong–Commit Mental Health Promotion Campaign

As described in Donovan and Anwar-McHenry [22], Act–Belong–Commit is an evidence-based comprehensive population-wide mental health promotion campaign with the objectives of building positive mental health and resilience, and preventing mental illness. The campaign utilises a community-based social franchising approach, supported by paid and unpaid main and social media messaging. The campaign targets individuals to encourage them to engage in mentally healthy activities, and simultaneously targets organisations that offer mentally healthy activities and supports them to promote and increase participation in their activities [23,24]. The campaign originated in Western Australia, where it is directed by Mentally Healthy WA (MHWA) at Curtin University, and is implemented through partnerships with health services, local governments, state government departments, workplaces, community organisations, and local clubs [23]. The campaign constructs appear to be universal and have been adopted and articulated in a local context in a number of countries around the globe, particularly in Scandinavian countries [25,26,27].

The Act–Belong–Commit constructs can be elaborated as follows to promote the message that individuals can build positive mental health and resilience by keeping physically, mentally, spiritually, and socially active (Act); keeping up contacts with friends and family and participating in community organisations and events (Belong); and being involved in challenges or causes that provide meaning and purpose in their lives (Commit). There is a considerable and increasing evidence base that these three behavioural constructs are associated with and contribute to both positive mental health and physical health [22,28,29].

### 1.2. The Act–Belong–Commit Mentally Healthy Schools Framework (MHSF)

As a result of schools expressing a desire to promote the Act–Belong–Commit mental health message within their schools, Act–Belong–Commit developed the Mentally Healthy Schools Framework (MHSF) [30]. The Mentally Healthy Schools Framework is based on the fundamental constructs of the Act–Belong–Commit general population campaign, adapted to the school setting via the World Health Organisation’s Health Promoting Schools Framework, which encourages a whole-of-school approach to mental health promotion through the three domains of the WHO’s Health Promoting Schools Framework: (i) Curriculum, Teaching and Learning; (ii) Partnerships and Services; and (iii) School Environment, Ethos and Organisation [31]. Schools who sign up as Act–Belong–Commit partners are provided with a variety of promotional resources that can be applied across the whole school community, including teachers and other staff. The program is self-sustaining and complements areas of the Australian curriculum, giving schools the flexibility to adapt the Framework to their specific requirements and priorities, and hence reducing the demands on staff. Participating schools sign an agreement with Act–Belong–Commit, and staff participate in training activities delivered by Act–Belong–Commit staff. Schools receive a variety of support resources, including a copy of the Mentally Healthy Schools Handbook, various communication materials, signage, and merchandise to assist with the delivery of the program (see https:///www.actbelongcommit.org.au, accessed 1 February 2023). These resources are provided to assist the incorporation of the Act–Belong–Commit constructs into the school curriculum and school policies and guidelines (for example, a student overall wellbeing policy, and specific health and physical education policies). By way of example, Act–Belong–Commit posters are placed in classrooms and around the school, the constructs of the campaign are included in curriculum components and referred to in school assemblies when appropriate to do so, and students engage in activities such as photovoice and developing television commercials or short videos to illustrate the positive mental health constructs of Act–Belong–Commit. The latter is a very popular activity and has become an annual competition between participating schools (for further implementation details and specific case studies, see https://www.actbelongcommit.org.au/programs-initiatives/mentally-healthy-schools/ (accessed 1 February 2023).

This paper describes a preliminary evaluation of the impact on students of the first phase of the Schools Program. An impact evaluation conducted concurrently on teachers is reported in Anwar-McHenry et al. [32].

## 2. Materials and Methods

Surveys of both teachers and students were undertaken using self-completion, structured questionnaires in the period 2016–2017. The procedures and methods are described in Anwar-McHenry et al. [32,33]. The focus in this paper is on the survey of students. 

### 2.1. Sample

Students aged 11–14 years in grades 7 to 9 from two schools that had adopted but not yet fully implemented the Act–Belong–Commit Mentally Healthy Schools Framework completed a “Baseline” questionnaire. Students in grades 7 to 9 from three schools that had been implementing the Act–Belong–Commit Mentally Healthy Schools Framework for 17 months or more completed a “Follow-up” questionnaire. Students were asked to obtain “active consent” from parents before being provided with a questionnaire. “Active consent” requires the student to take a consent form home, ask the parent to read and sign it if they approve, and return the signed form to the school. Hence, the number of students completing a questionnaire in the allowed time frame (N = 140) was understandably limited. As an incentive for participation, those completing the questionnaire were offered the opportunity to be entered into a draw to win a prize. 

### 2.2. Questionnaires

The “Baseline” questionnaire for students in schools that had not yet fully implemented the Act–Belong–Commit Framework focussed on the student’s prior awareness of the Act–Belong–Commit campaign in the community and their understanding of the campaign messages. They were also asked whether they had tried to do something as a result of their exposure to those messages, and their perceived impact of the campaign in the community. 

The “Follow-up” questionnaire in participating schools focussed on school activities under the Act–Belong–Commit initiative and included questions as to students’ awareness of their school’s involvement in the Act–Belong–Commit campaign, and whether they were aware of and had participated in specific Act–Belong–Commit activities at their school. They were also asked whether they had tried to do something for their mental health because of their exposure to those messages, and their perceived impact of the campaign in the school. Both the baseline and follow-up questionnaires included measures of school belonging and the extent to which the teachers encouraged various physical and mental health activities (reported in Anwar-McHenry et al., [33]). 

The preliminary impact questions reported here include:

(i) Students’ awareness of the Act–Belong–Commit campaign, and, for follow-up students, awareness of the school’s involvement in the Act–Belong–Commit campaign;
(ii) Students’ understanding of the message of the Act–Belong–Commit campaign;
(iii) Whether baseline and follow-up students aware of the campaign had tried to do something for their mental health as a result of their exposure to the campaign;
(iv) Whether follow-up students had talked about the campaign and mental health with other students and their family;
(v) Whether follow-up students had changed the way they think about mental health as a result of the campaign;
(vi) Whether follow-up students believed that the campaign at the school had increased openness about mental health (see [33] for complete baseline and follow-up questionnaires).

This research was granted ethics approval from Curtin University’s Human Research Ethics Committee (Approval RDHS-216-15) and the Department of Education (Approval D16/0023499).

## 3. Results

A total of 140 students across the three years completed either a baseline (n = 90) or follow-up questionnaire (n = 50), with a majority being female students at both baseline (61%) and follow-up (67%), and a higher proportion of grade 9 students (46%) than grades 7 and 8 (27% each).

### 3.1. Campaign Awareness 

Both baseline and follow-up respondents were asked: “Have you heard of the Act–B–long–Commit campaign?”. Awareness of the campaign amongst follow-up students was almost 90%, which was significantly higher than amongst baseline students (86% vs. 62% respectively) (chi-square, *p* < 0.02), indicating that the Act–Belong–Commit intervention at the school substantially increased student awareness of the campaign. 

Both baseline and follow-up students who reported being aware of the campaign were provided with the possible sources listed in Table 1 and were asked to indicate all sources “where they had heard about the campaign”. Consistent with the greater campaign awareness overall amongst follow-up students, more follow-up students nominated “at school” as a source of campaign awareness relative to baseline students: 86% vs. 51% (*p* < 0.01). 

### 3.2. Campaign Understanding 

The baseline and follow-up students who were aware of the campaign were asked “what Act–Belong–Commit means and what the campaign is trying to do”. Their responses are shown in Table 2, with “Act”, “Belong” and “Commit” responses categorised as such. Overall, Table 2 shows that both baseline and follow-up students aware of the campaign demonstrate a good understanding of the campaign messages and what the campaign is trying to achieve, both in terms of acting, belonging, and committing activities, as well as the positive messages about mental health. It is also of note that 23% of baseline and 15% of follow-up students nominated help seeking for mental health problems.

### 3.3. Campaign Behavioural Impact—Acted on the Campaign Messages

All baseline and follow-up students who reported being aware of the Act–Belong–Commit campaign were asked whether they “had done or tried to do something as a result of becoming aware of the Act–Belong–Commit message”. Of those students aware of the campaign, slightly (but not significantly) more follow-up than baseline respondents reported trying to do something for their mental health as a result of the campaign: 30% vs. 25%. Given that awareness was substantially higher in follow-up versus baseline respondents, this moderate increase in awareness nevertheless translates into a substantially greater number of students overall being stimulated by the campaign to take actions to enhance their mental health: 26% of all follow-up students versus 15% of all baseline students.

### 3.4. Campaign Behavioural Impact—Talked with Others about Mental Health or the Campaign

Follow-up students aware of the campaign were asked whether the campaign had stimulated them to talk about mental health with their school friends and with their family, and whether they had talked specifically about the Act–Belong–Commit campaign with their school friends and their family. Their responses are shown in Table 3. 

With respect to talking about mental health, Table 3 shows that the campaign has stimulated about one in four students to talk with school friends about mental health (24%), but less so with family (14%). With respect to talking specifically about the Act–Belong–Commit campaign, 10% reported talking about the campaign with school friends and 14% with family. 

### 3.5. Campaign Impact on How Follow-Up Students Think about Mental Health

Follow-up students who were aware of the campaign were asked whether they “had changed the way they think about mental health as a result of the Act–Belong–Commit message”. Just over a third (37%) of follow-up students responded “yes”. 

Those who answered “yes” were asked an open-ended question: “in what way?” The responses to this question primarily referred to an increased awareness and empathy about mental health (just over half of respondents) and their taking up activities for their mental health (one in five respondents). This suggests that the Act–Belong–Commit message encourages a positive and proactive view of mental health as distinct from stimulating mental illness connotations.

### 3.6. Campaign Impact on Openness around Mental Health

Follow-up respondents aware of the campaign were asked whether “the campaign had made students more open about mental health issues, less open, or made no difference”. Their responses are shown in Table 4. 

Table 4 shows that almost one in four (23%) follow-up students believed the campaign had made students more open about mental health issues, with around the same number stating “no difference” (26%). None of the follow-up students responded, “less open”. However, almost half (51%) responded “don’t know/can’t say” to this question.

### 3.7. Follow-Up Students’ Awareness of and Participation in Act–Belong–Commit Activities in the School 

Follow-up students who were aware of the campaign were asked whether they “had participated in any Act–Belong–Commit events at their school”. They were also asked whether “their teachers or principal had talked about the campaign to students”, and, if yes, were given the response categories shown in Table 5 and were asked “how often they saw or heard something about Act–Belong–Commit at their school”. 

Of the follow-up students aware of the campaign, almost half (49%) stated that they had participated in an Act–Belong–Commit event at their school, and almost three-quarters (72%) stated that their teachers or principal had talked about Act–Belong–Commit. 

Amongst those reporting that their teachers or principal had talked about the campaign, Table 5 shows that one-third (33%) reported seeing or hearing about Act–Belong–Commit every two weeks or more, around one-third (35%) reported monthly, and around one-third (32%) reported less than monthly. 

## 4. Discussion

Given that the Act–Belong–Commit campaign was being promoted in the general community at that time, almost two-thirds of the baseline students (62%) reported awareness of the Act–Belong–Commit campaign. This awareness increased substantially amongst follow-up students to almost 9 in 10 students (86%). Further, those aware of the campaign reported understandings of the campaign that were consistent with the specific campaign messages (acting, belonging, committing) and/or generally positive associations with and implications for mental health. It is of note that when asked whether they were aware of the campaign in their school, 51% of baseline students stated they were; it appears that the recent announcement of the school adopting the Act–Belong–Commit Mentally Healthy Schools Framework had some impact on awareness at the school.

Given that almost half of follow-up students had participated in an Act–Belong–Commit event at their school, and almost three-quarters reported their teachers or principal talking about Act–Belong–Commit, it appears that the participating schools have been relatively successful in engaging students in their mental health promotion activities, and, perhaps more importantly, that staff at these schools were actively participating in intervention activities and promoting the messages to their students. Given the importance of teachers as role models for students, this is a very important finding (for example, Cheung [34] found that in physical education classes, students were more physically active if their teachers were also more physically active). Nevertheless, the results with respect to frequency of observing Act–Belong–Commit-related activities at their school not only indicate some variability in the extent of campaign activities in these schools, but overall indicate that campaign activities could be increased.

Act–Belong–Commit surveys of the general adult population show that 10–15% of adults aware of the campaign have tried to do something for their mental health as a result of exposure to the campaign. The percentage of baseline students was 25%, which may reflect an additional initial impact of the announcement of the campaign in those schools. A slightly, but not significantly, higher percentage of students at follow-up reported trying to do something for their mental health as a result of their exposure to the campaign (30%). Of note is that 43% of teachers at follow-up versus 21% of teachers at baseline reported doing something for their mental health as a result of the campaign. These data indicate that implementation of a community-wide campaign in a specific setting can have a substantially increased impact on the behaviour of those exposed to the campaign.

As in the general population evaluations [35,36], the results show that the Act–Belong–Commit Mentally Healthy Schools Framework intervention is encouraging students and staff to talk about mental health and/or the Act–Belong–Commit campaign with friends and family. This facilitation of talking about mental health is consistent with general population findings that the campaign is believed to increase openness about mental health and to decrease stigma around mental illness, which together have implications for increased early help seeking [37], and hence the prevention of more serious mental health problems. 

The finding that small but moderate percentages of students believe that the Act–Belong–Commit Framework encourages help seeking is a very positive outcome supporting a reduction in stigma associated with such help seeking. This outcome is noteworthy given that stigma around mental illness constitutes a major barrier to seeking help for a mental health problem [38,39,40]. 

A substantial proportion of follow-up students (37%) reported changing the way they think about mental health, and in positive ways such as an increased awareness about mental health, an increased importance placed on mental health, and their taking up activities for their mental health. It is noted that these changes are consistent with increased openness around mental health and decreased stigma around mental illness.

Overall, the findings reported here suggest that the Mentally Healthy Schools Framework intervention has not only increased awareness of the Act–Belong–Commit campaign in students but has also increased their mental health literacy and engagement in behaviours that contribute to positive mental health. These data support the conclusion that this Act–Belong–Commit school intervention could contribute significantly to students engaging proactively in activities to strengthen and maintain their mental health, which would have substantial positive individual and societal impacts as this age group matures. 

The above findings are similar to those found on the impact of the Act–Belong–Commit Mentally Healthy Schools Framework intervention on teachers, where substantial proportions of teachers reported acting on the campaign’s messages, talking more about mental health with friends, family, and other school staff, changing the way they thought about mental health, and believing that the campaign decreased stigma around mental illness and increased openness about mental health issues [32]. It is likely that whole-school interventions such as the Act–Belong–Commit Mentally Healthy Schools Framework that are delivered by teaching staff rather than mental health professionals have a more positive impact because the teaching staff themselves act on the messages and hence act as positive mental health role models for the children. Overall, these findings contribute to Cavioni’s [21] call for school mental health interventions to include both students and staff and confirm that whole-school approaches are associated with more positive outcomes than more limited scope interventions [41,42,43]

The positive impact of the Act–Belong–Commit Mentally Healthy Schools Framework on students is also consistent with Liao et al.’s [15] systematic review of school mental health intervention experiments led by teachers. Liao et al. [15] found that the reviewed interventions had a significantly positive impact on increased mental health literacy, and also reduced stigma around mental illness, at least in the short term. However, they found no impact on willingness to seek help. It may well be that sustained interventions such as the Act–Belong–Commit Mentally Healthy Schools Framework would not only increase literacy and reduce stigma, but could also increase help seeking amongst students as the community-wide campaign does in the general population [37]. 

The primary limitation of this study is the relatively small number of participants in the baseline and follow-up surveys. Future studies should increase the number of participants across the three grades (7–9), and also include measures of impact on the students’ mental health and wellbeing, and the impact on help seeking.

## 5. Conclusions

Overall, the above findings show a positive intervention impact on (i) students’ mental health literacy, (ii) students’ openness about mental health, (iii) how students think about mental health, and (iv) stimulating students’ engagement in behaviours conducive to good mental health. It is noted that these impacts are consistent with positive findings for whole-school interventions [42] and where teachers deliver the intervention rather than mental health professionals [15]. However, these positive effects on students require confirmation in a larger sample and over a longer time period. 

Nevertheless, although based on a limited sample size, the findings of this preliminary evaluation on students, in conjunction with the contemporaneous findings of a positive impact on staff [32], and previous positive process evaluation effects [30], suggest that the Act–Belong–Commit Mentally Healthy Schools Framework could have a considerable positive impact on the mental health of both staff and students. Such impacts would have positive flow-on effects for the whole of society as each student cohort moves into employment and adulthood. These staff and student findings also indicate that school-based interventions could be enhanced by an accompanying general population campaign, and, conversely, that community-wide mental health promotion campaigns could benefit considerably by engaging institutions such as schools, worksites, and community organisations to conduct mental health promotion activities within their jurisdictions. 

## Figures and Tables

**Table 1:** Sources of awareness of Act–Belong–Commit.

	% * Baseline Aware	% * Follow-Up Aware (n = 43)
	(n = 56)	
At school	51	86
Television	70	56
Community events	32	28
Newspaper	19	5
At local club	8	5
Other	0	14
No response	0	0

* Totals exceed 100% as multiple responses were permitted.

**Table 2:** Students’ understanding of the Act–Belong–Commit campaign *.

	% Baseline Aware (n = 56)	% Follow-Up Aware (n = 43)
Act: stay active, take part in things, encourage others to act	17%	10%
Belong: be a part of community/group, bring people together	31%	39%
Commit: commit to things, commit to a cause	13%	12%
Be healthier, keep physically and/or mentally healthy, make people happy	35%	44%
What to do if you need help, encourage help seeking, help with health problems, awareness raising	23%	15%
Be kind, make students feel safe, friendly/safe environment	-	10%
Make good decisions, prepare for future, achieve greatness	6%	-
Reduce stigma, anti-bullying	4%	-
Don’t know, unsure	10%	12%

* Totals exceed 100% as multiple responses were permitted.

**Table 3:** Campaign impact on talking about mental health or the campaign with school friends and family: % of follow-up students aware of Act–Belong–Commit (n = 43).

	% Talked about Mental Health	% Talked about A-B-C Campaign
With school friends		
Yes	24	10
No	74	88
No response	2	2
With family		
Yes	14	14
No	83	83
No response	2	2

**Table 4:** Perceived campaign impact on follow-up students’ openness about mental health issues.

	% Impact on Follow-Up Students’ Openness (n = 43)
More open	23
No difference	26
Less open	0
Don’t know/can’t say	51
	100%

**Table 5:** Follow-up respondents’ reporting of how often they saw/heard about Act–Belong–Commit at their school.

	% Teacher/Principal Talked about ABC
**Frequency**	**(n = 31) **
Once a week	7
Every 2 weeks	26
Once a month	35
Less than monthly	32
Total	100%

## Data Availability

Contact corresponding author for available data.
